# Supplementary material for: A niobium and tantalum co-doped perovskite cathode for solid oxide fuel cells operating below 500 °C
Source: Nat Commun. 2017 Jan 3;8:13990. doi: 10.1038/ncomms13990 (PMC5216129; doi:10.1038/ncomms13990)
Supplement: Supplementary Information — Supplementary Figures, Supplementary Tables and Supplementary References [file ncomms13990-s1.pdf]

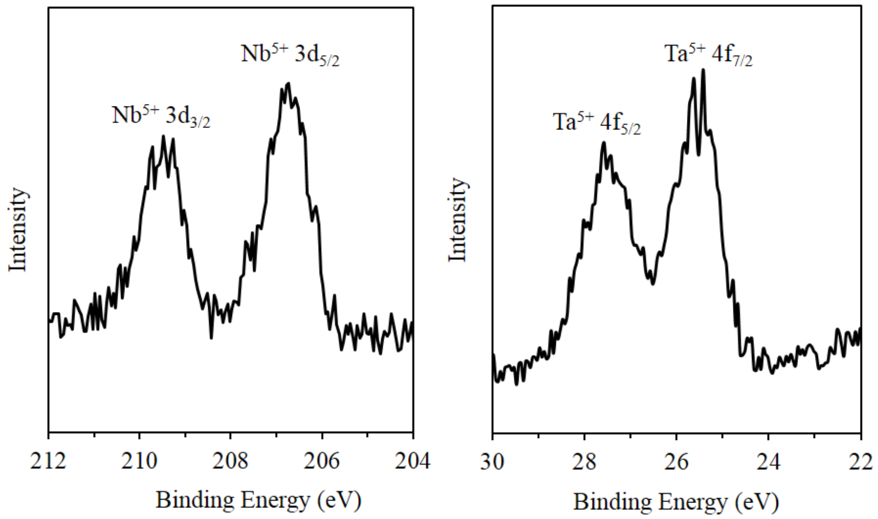

Supplementary Figure 1 X-ray photoelectron spectroscopy profile of Nb and Ta of SCNT at room temperature.

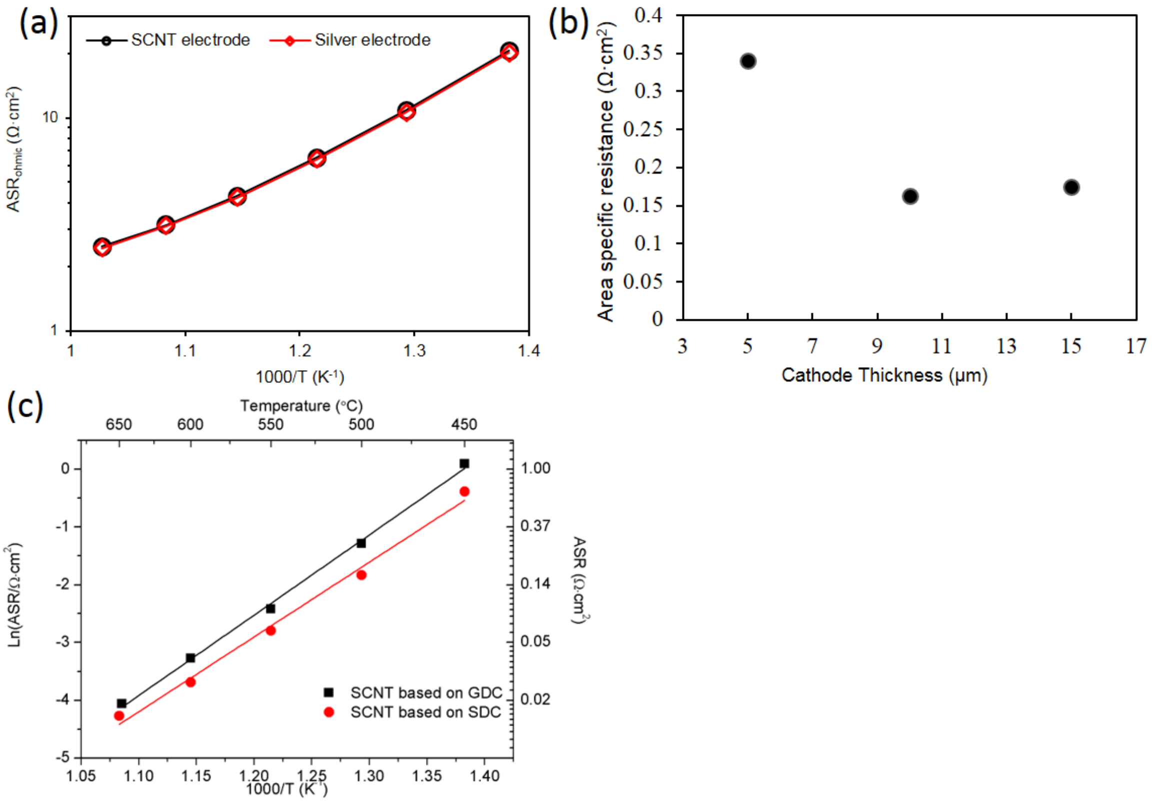

Supplementary Figure 2 Factors that may affect the area specific resistance of SCNT cathode. (**a**) A comparison of ohmic resistances between a GDC-based symmetrical cell with SCNT cathode and a cell with silver electrode (similar electrolyte thickness to the SCNT one). The cell with silver electrode was fabricated by applying silver paste to both sides of the GDC electrolyte pellet followed by solidifying at 260 °C. (**b**) ASRs of SCNT cathodes with different cathode thicknesses. (**c**) ASRs of SCNT cathode in SDC and GDC-based symmetrical cells as a function of temperature.

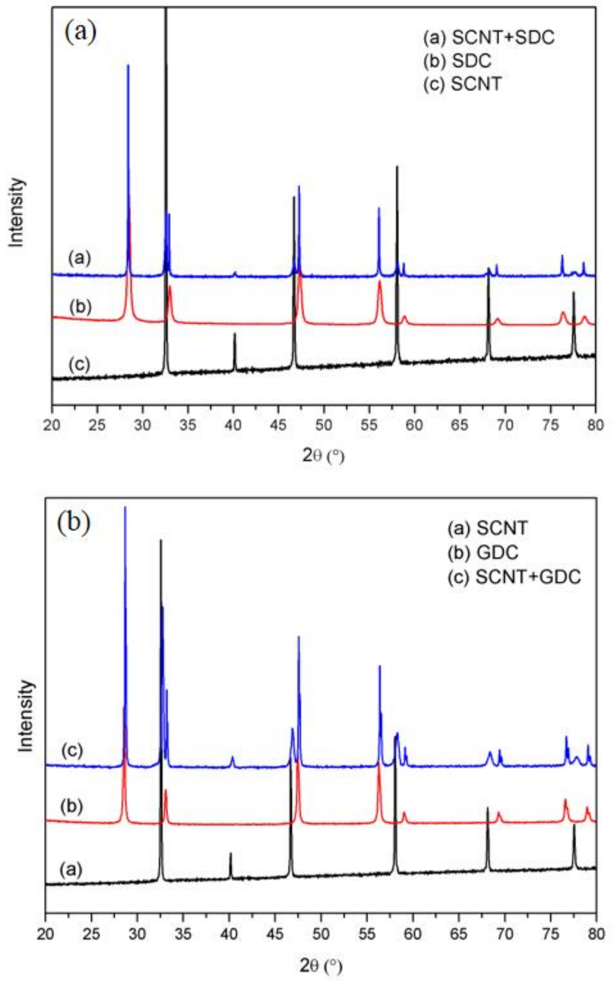

Supplementary Figure 3 Compatibility of SCNT materials with different electrolytes. X-ray diffraction data of (**a**) pure SCNT, SDC, and a 50:50 wt% SCNT/SDC mixture after 2h-sintering at 1000 ºC; (**b**) pure SCNT, GDC, and a 50:50 wt% SCNT/GDC mixture after 2h-sintering at 1000 ºC. The mixture was made by mechanically mixing the powders SCNT and SDC (or GDC) at room temperature.

Cathode Material Compositions	Area specific resistance ($\Omega\cdot cm^2$)	Reference
$SrCo_{0.8}Nb_{0.1}Ta_{0.1}O_{3-\delta}$	~0.16 @500°C<br>~0.68 @450°C	This work
$Ba_2Bi_{0.1}Sc_{0.2}Co_{1.7}O_{6-\delta}$	~1.50 @ 500°C	1
$SrSc_{0.175}Nb_{0.025}Co_{0.8}O_{3-\delta}$	~0.32 @500°C	2
$NdBa_{0.75}Ca_{0.25}Co_{0.25}Co_2O_{5+\delta}$	~0.67 @500°C	3
$Ba_{0.9}Co_{0.7}Fe_{0.2}Mo_{0.1}O_{3-\delta}$	~0.28 @500°C<br>~1.09 @450°C	4
$Ba_{0.5}Sr_{0.5}Co_{0.8}Fe_{0.2}O_{3-\delta}$	~0.50 @500°C	5
$SrSc_{0.2}Co_{0.8}O_{3-\delta}$	~0.45 @500°C	6
icn-$La_{0.8}Sr_{0.2}MnO_3$+$Bi_{1.6}Er_{0.4}O_3$	~0.61 @500°C	7
$PrBa_{0.5}Sr_{0.5}Co_{1.5}Fe_{0.5}O_{5+\delta}$	~0.33 @500°C	8

Supplementary Table 1 Comparison of ASR values between SCNT and other highly active cathode compositions in literatures

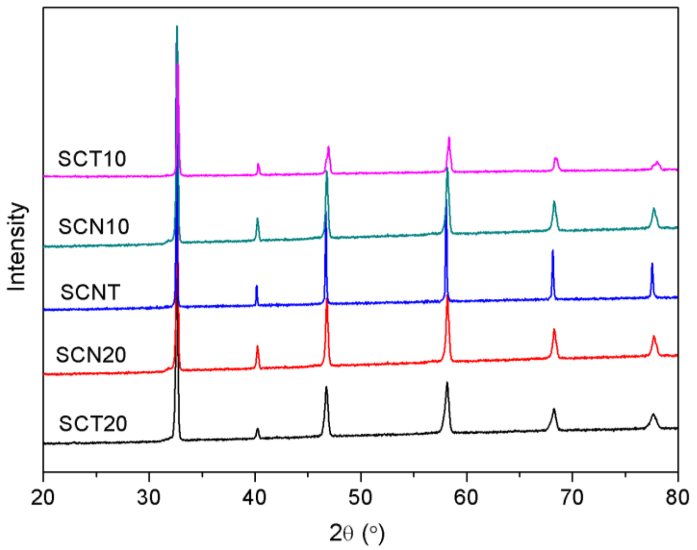

Supplementary Figure 4 X-ray (CuK_{α}) diffraction patterns of SCNT, SCN20, SCT20, SCN10 and SCT10 at room temperature.

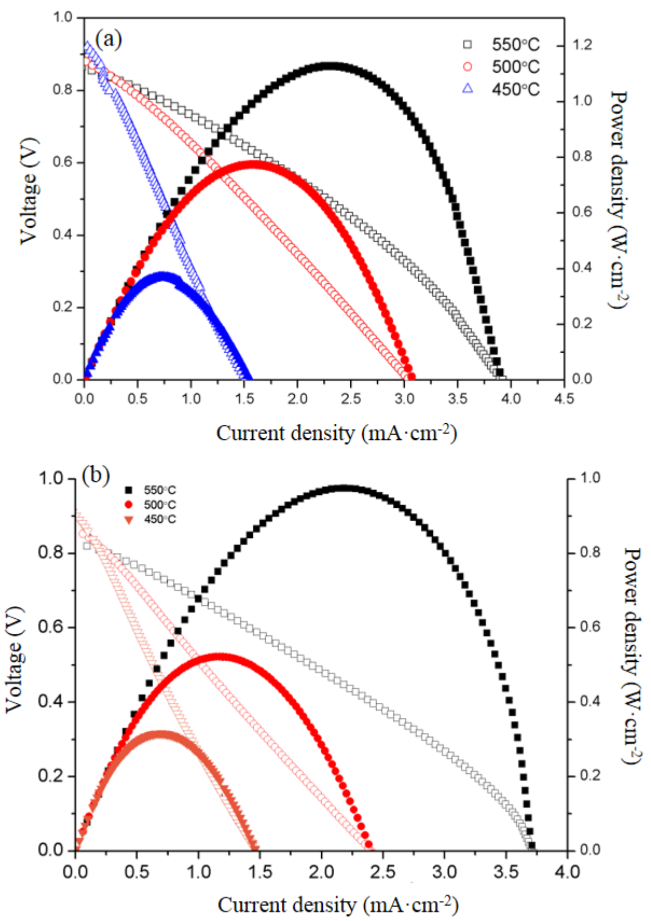

Supplementary Figure 5 Performance of single fuel cell with SCNT or BSCF cathode. (**a**) Single-cell performance of a SCNT| SDC (~20μm)| Ni+SDC cell. (**b**) Single cell performance of a BSCF|GDC(~14μm) | Ni+GDC cells showing a performance that is similar to that reported for BSCF-based SOFCs.[5, 9]

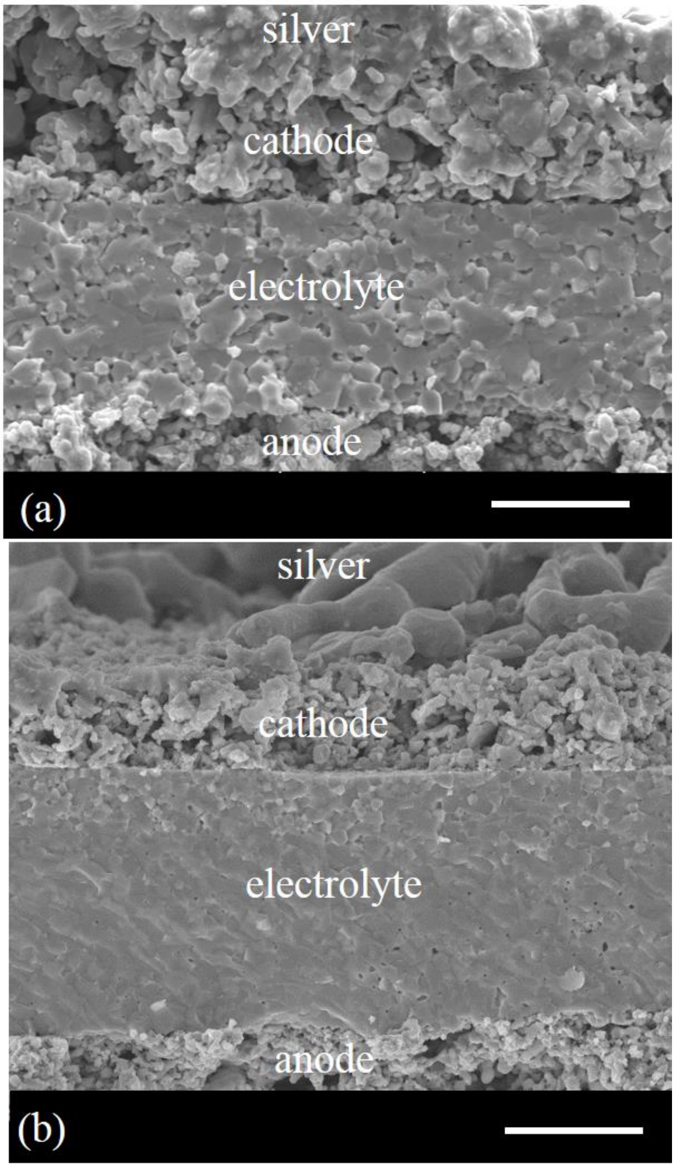

Supplementary Figure 6 Micrographs of cross-sections of single cell with SCNT cathode. SEM image of (**a**) an anode-supported single cell with GDC electrolyte (~ 14 μm) and SCNT cathode and (**b**) the SCNT-based single cell. (electrolyte thickness ~ 20 μm). Scale bar is 10 μm.

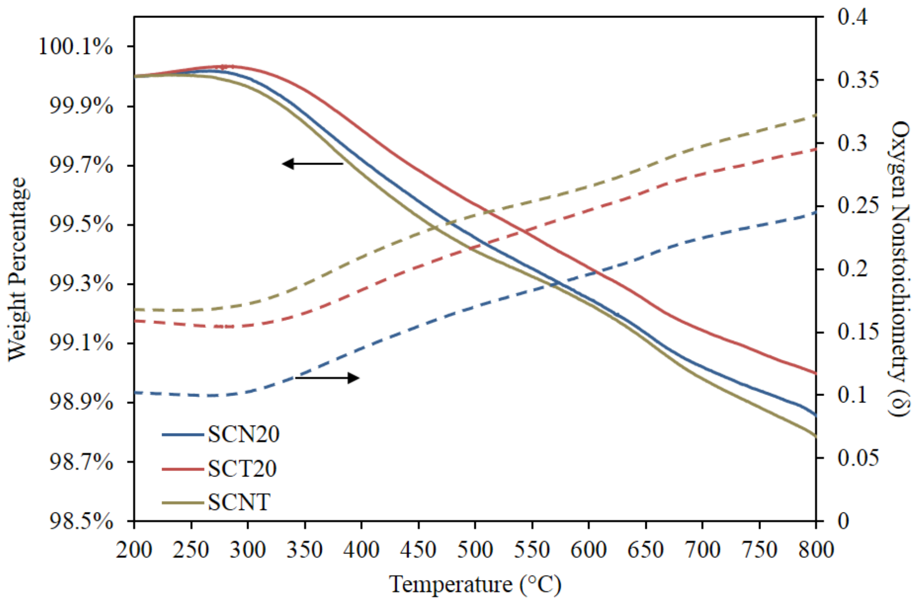

Supplementary Figure 7 Study of oxygen vacancy content of single-doped and co-doped materials at different temperature. Mass and oxygen nonstoichiometry change of SCN20, SCT20 and SCNT as a function of temperature in the flowing air with a flow rate of 20mL·min^{-1}.

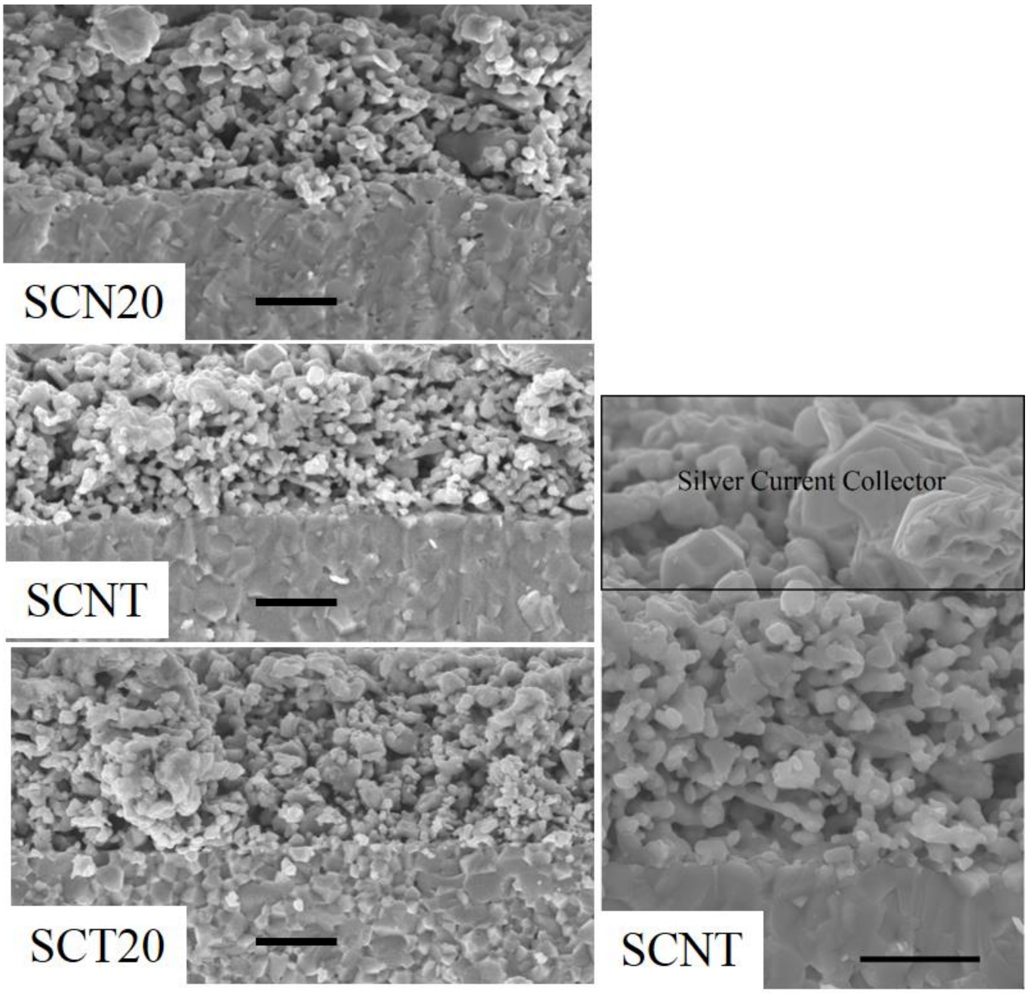

Supplementary Figure 8 Micrographs of cross-sections of cathode materials. Cross sectional SEM images of SCNT, SCN20, and SCT20 cathodes in a symmetrical cell configuration, and an example of cross section of SCNT cathode with silver paste. Scale bar is 5 μm.

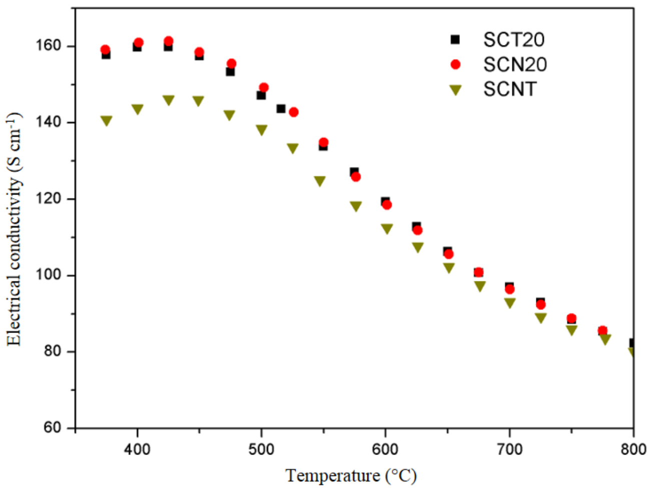

Supplementary Figure 9 Electrical conductivity of SCN20, SCT20, and SCNT samples using 4-probe DC method as a function of temperature.

	①-②	②-③	③-④	④-⑤
**$SrCo_{0.75}Nb_{0.25}O_{3-\delta}$**	**0.433**	0.322	0.406	**0.433**
**$SrCo_{0.75}Ta_{0.25}O_{3-\delta}$**	**0.638**	0.442	0.525	**0.638**
**$SrCo_{0.75}Nb_{0.125}Ta_{0.125}O_{3-\delta}$**	**0.572**	0.323	0.437	0.228

Supplementary Table 2 The calculated energy barriers (eV) of an oxygen vacancy (V_O) migrating along the pathway shown in Figure 3(b) in Nb-, Ta-, and Nb/Ta-doped models.

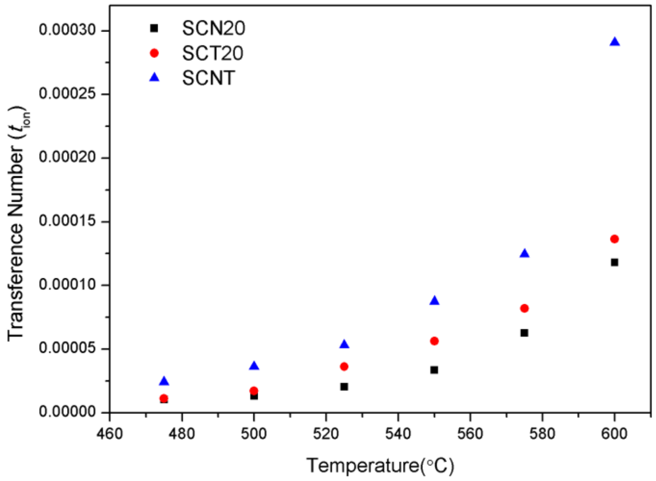

Supplementary Figure 10 Ionic transference numbers (t_{ion}) as a function of temperature for SCN20, SCT20 and SCNT.

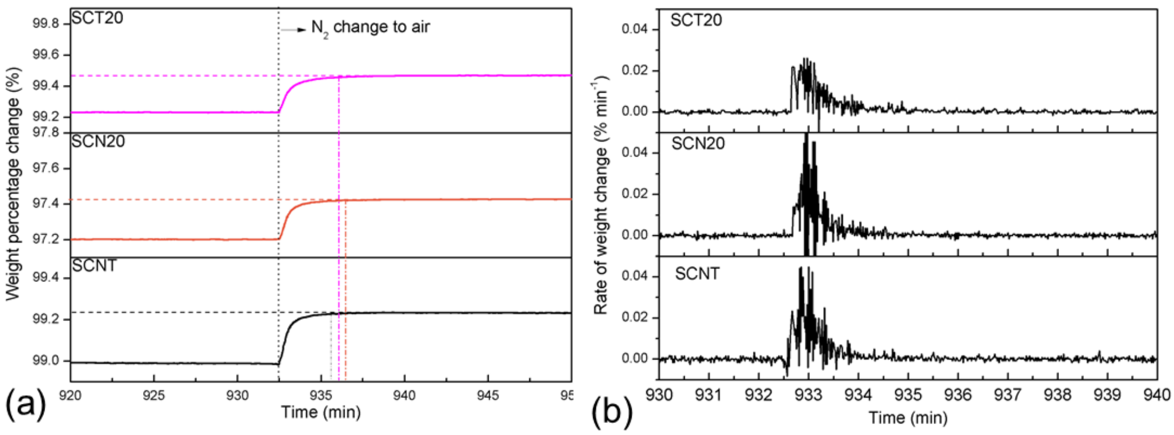

Supplementary Figure 11 Response of single-doped and co-doped materials to oxygen partial pressure changes. (**a**) Percentage weight change and (**b**) the corresponding rate of weight change, of SCN20, SCT20, and SCNT heated to 500 °C during a change of atmosphere from flowing nitrogen to flowing air. The weight change is a result of the intake of oxygen into the sample from the ambient air, with SCNT reaching equilibrium in the shortest time (~ 188 s) compared with SCN20 (~ 245 s) and SCT20 (~ 217 s).

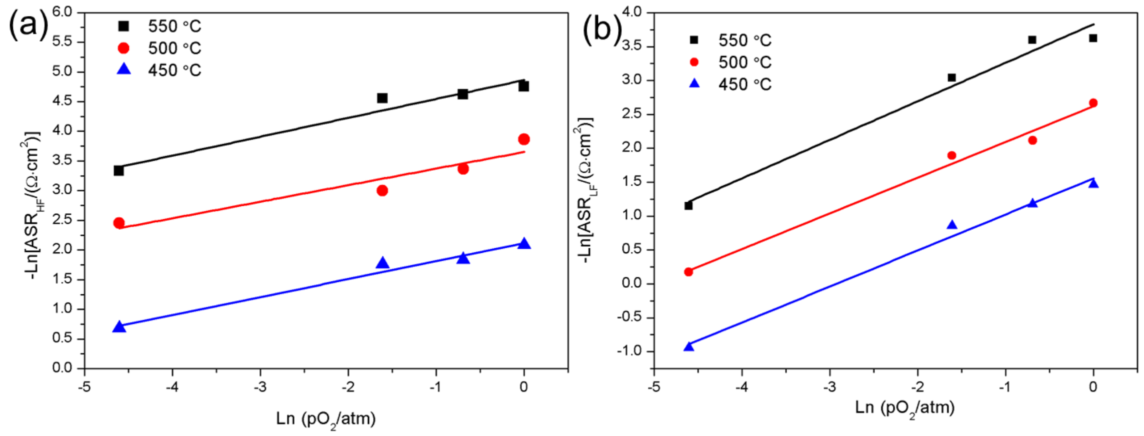

Supplementary Figure 12 Mechanism study on SCNT cathode at different temperature and oxygen partial pressures. ASR values of SCNT cathode corresponding to processes at (**a**) high frequencies and (**b**) frequencies as a function of pO_2 from 550°C to 450 °C. The slope m for $-\ln(ASR_{HF})\sim\ln(pO_2)$ is 0.28-0.32, which is close to 0.25, indicating that the process at high frequencies is related to charge-transfer process to the absorbed oxygen species. The slope for low frequencies is 0.53-0.57, suggesting non-charge-transfer process.[10]

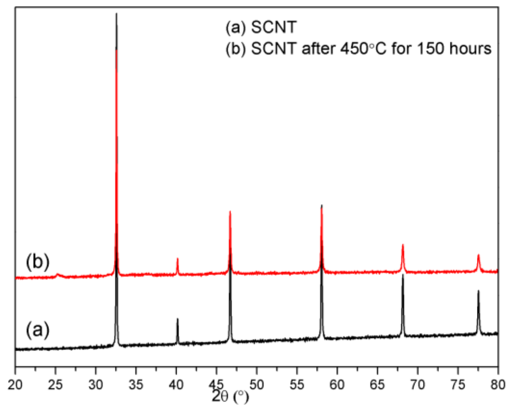

Supplementary Figure 13 XRD patterns of SCNT before and after heat treatment at 450 °C for 150 h.

Supplementary References

1. Zhou W, *et al.* Novel B-site ordered double perovskite $Ba_2Bi_{0.1}Sc_{0.2}Co_{1.7}O_{6-x}$ for highly efficient oxygen reduction reaction. *Energy & Environmental Science* **4**, 872-875 (2011).

2. Zhou W, Sunarso J, Zhao M, Liang F, Klande T, Feldhoff A. A Highly Active Perovskite Electrode for the Oxygen Reduction Reaction Below 600 °C. *Angewandte Chemie International Edition* **52**, 14036-14040 (2013).

3. Yoo S, *et al.* Development of Double-Perovskite Compounds as Cathode Materials for Low-Temperature Solid Oxide Fuel Cells. *Angewandte Chemie International Edition* **53**, 13064-13067 (2014).

4. Huang S, Lu Q, Feng S, Li G, Wang C. $Ba_{0.9}Co_{0.7}Fe_{0.2}Mo_{0.1}O_{3-\delta}$: A Promising Single-Phase Cathode for Low Temperature Solid Oxide Fuel Cells. *Advanced Energy Materials* **1**, 1094-1096 (2011).

5. Shao Z, Haile SM. A High-Performance Cathode for the Next Generation of Solid-Oxide Fuel Cells. *Nature* **431**, 170-173 (2004).

6. Zhou W, Shao Z, Ran R, Cai R. Novel $SrSc_{0.2}Co_{0.8}O_{3-\delta}$ as a Cathode Material for Low Temperature Solid-Oxide Fuel Cell. *Electrochemistry Communications* **10**, 1647-1651 (2008).

7. Lee KT, Lidie AA, Yoon HS, Wachsman ED. Rational Design of Lower-Temperature Solid Oxide Fuel Cell Cathodes Via Nanotailoring of Co-Assembled Composite Structures. *Angewandte Chemie International Edition* **53**, 13463-13467 (2014).

8. Choi S, *et al.* Highly Efficient and Robust Cathode Materials for Low-Temperature Solid Oxide Fuel Cells: $PrBa_{0.5}Sr_{0.5}Co_{2-X}Fe_XO_{5+\delta}$. *Scientific reports* **3**, (2013).

9. Liu QL, Khor KA, Chan SH. High-performance Low-temperature Solid Oxide Fuel Cell with Novel BSCF Cathode. *Journal of Power Sources* **161**, 123-128 (2006).

10. Takeda Y, Kanno R, Noda M, Tomida Y, Yamamoto O. Cathodic Polarization Phenomena of Perovskite Oxide Electrodes with Stabilized Zirconia. *J Electrochem Soc* **134**, 2656-2661 (1987).
